# Elevated metabolic score for visceral fat was associated with increased prevalence of gallstones in American adults: a cross-sectional study

**DOI:** 10.3389/fmed.2024.1474368

**Published:** 2024-11-07

**Authors:** Hao Lin, Kexuan Shi, Shuang Luo, Wu Ye, Xiaoniao Cai

**Affiliations:** ^1^Department of Gastroenterology, Pingyang Hospital of Wenzhou Medical University, Wenzhou, China; ^2^Department of Emergency Medicine, Pingyang Hospital of Wenzhou Medical University, Wenzhou, China

**Keywords:** insulin resistance, obesity, METS-VF, gallstones, visceral adipose tissue

## Abstract

**Background:**

Metabolic Visceral Fat Score (METS-VF) recently introduced is posited to be a superior metric for assessing visceral adipose tissues (VAT) compared to traditional obesity indexes. This study aims to elucidate the correlation between METS-VF and the incidence of gallstones.

**Methods:**

In this cross-sectional study, the data from the National Health and Nutrition Examination Survey (NHANES) during the period from 2013 to 2020 were analyzed. And the correlation between METS-VF and the incidence of gallstones was explored through multivariate logistic regression analysis, receiver operating characteristic (ROC) curve, subgroup analysis and restricted cubic spline (RCS) regression.

**Results:**

This study included 5,975 participants, of whom 645 (10.8%) were gallstone formers. As the quartile range of METS-VF increased, a notable rise in the prevalence of gallstones was observed (3.2% vs. 7.4% vs. 12.1% vs. 20.6%, *p* < 0.001). Logistic regression analyses indicated a significant positive correlation between METS-VF and the risk of gallstones (OR = 3.075, 95% CI: 2.158, 4.381). Subgroup analyses further revealed a stronger correlation between gallstones and METS-VF in subjects over 50 years old. RCS regression identified a non-linear positive correlation, with an inflection point at 6.698. Finally, the area under the ROC curve (AUC) of METS-VF was significantly larger (AUC = 0.705, 95%: 0.685, 0.725) than those of traditional obesity indexes and other VAT surrogate markers.

**Conclusion:**

This study is the first to reveal a significant positive correlation between the prevalence of gallstones and METS-VF, with METS-VF outperforming other VAT surrogate markers in the diagnosis of gallstones.

## Introduction

Gallstones are one of the most prevalent digestive diseases worldwide, and risk factors have been well established for gallbladder cancer as well (1, 2). Gallstones represent a substantial healthcare burden in the United States, impacting up to 15% of Americans (3, 4). Epidemiological data indicated that the prevalence of gallstones ranges from 10 to 15% among adult Caucasians. which can be as high as 70% among American Indians (5, 6). While gallstones are typically asymptomatic, 10 to 25% of affected individuals may experience specific symptoms such as acute cholecystitis and biliary pain. Among these symptomatic cases, 1 to 2% may develop severe complications (1, 7–9), which can result in significant pain and potentially life-threatening conditions. Although previous studies have identified risk factors correlated with the formation of gallstones, there is still an absence of dependable clinical indexes for the prevention of gallstones.

Pregnancy, female, race, and age over 40 years old are non-modifiable risk factors for the development of gallstones, each of which increases the risk of gallbladder by 4 to 10 times (3, 4). Among modifiable risk factors, metabolic syndrome, characterized by dyslipidemia, obesity, insulin resistance, and type 2 diabetes mellitus (T2DM), emerges as the most significant contributor to the development of gallstones (10). Obesity, particularly abdominal obesity, affecting approximately 25% of the population, is significantly correlated with the incidence of gallstones (3). Numerous studies have identified obesity as a risk factor for developing gallstones (11–13), with evidence indicating that the incidence of gallstones increased by a factor of 1.63 for every five-unit increment in body mass index (BMI) (12). Despite the strong correlation between obesity and the formation of gallstones, there is still a notable deficiency in reliable obesity indexes for predicting and assessing the risk of gallstones.

Studies have indicated that VAT exhibits a stronger correlation with metabolic diseases compared to subcutaneous fat (14–16). In a recent study, Bello-Chavolla et al. (17) found that METS-VF is significantly better than traditional obesity indexes in estimating VAT. METS-VF encompasses waist to height ratio (WHtR), BMI, hig-density lipoprotein cholesterol (HDL-C), fasting plasma glucose (FPG), triglycerides (TG), gender, and age. It can offer a comprehensive assessment of the metabolic impact and content of VAT. It can not only evaluate the distribution and content of glycolipid metabolism and body fat, but also incorporate gender and age differences in VAT. Recent studies have established METS-VF as a more effective predictor and assessor of metabolic disease risk, including hyperuricemia, hypertension, chronic kidney dysfunction (CKD), and T2DM, compared to traditional obesity indexes (18–22). Nevertheless, the correlation between gallstones and METS-VF, along with its potential utility in identifying individuals at increased risk for gallstones, remains undocumented.

Therefore, this study aims to evaluate the correlation between METS-VF and the prevalence of gallstones, and to compare the predictive value of METS-VF for gallstones with that of other VAT indexes (WHtR, visceral adiposity index (VAI), BMI, lipid accumulation product (LAP)), and IR related indexes (metabolic score for insulin resistance (METS-IR), triglyceride-glucose index (TyG)) within Americans.

## Methods

### Research subjects

The author obtained data from the National Health and Nutrition Examination Survey (NHANES),^1^ a national population-based cross-sectional study conducted by the National Center for Health Statistics (NCHS) to explore health status in Americans (23). The survey is conducted every 2 years by taking physical examinations, interviews, and various sections covering dietary, demographic, examination, and laboratory data.

**Figure 1:**
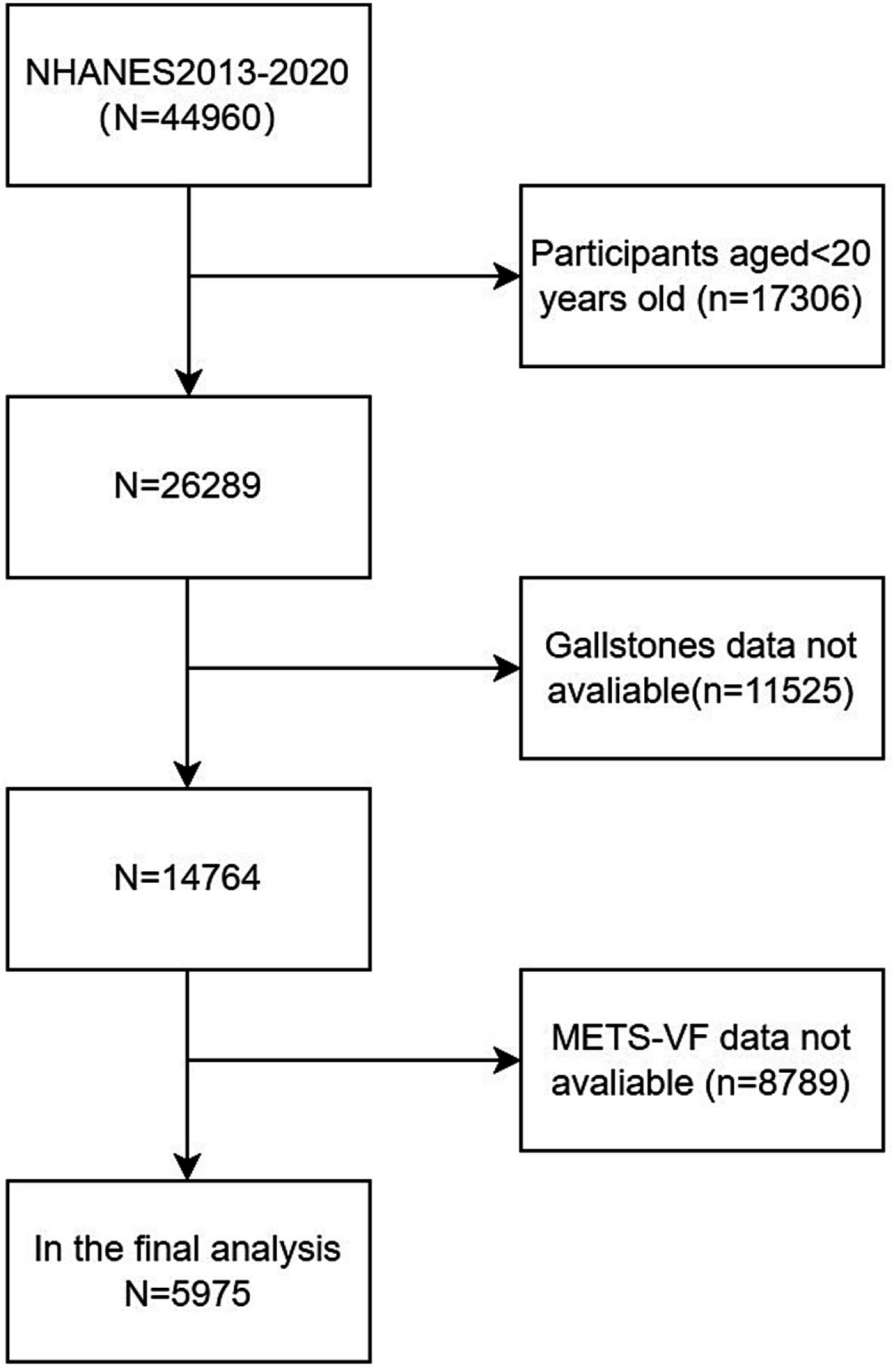
Flowchart of the sample selection from the 2013–2020 NHANES.

The baseline clinical data analyzed in this study were derived from NHANES 2013–2020. The data from subjects explicitly responding to questions regarding the presence of gallstones were included. A total of 44,960 participants completed the questionnaire. After excluding participants aged<20 years old (*n* = 17,306), missing data about gallstones (*n* = 11,525) and METS-VF (*n* = 8,789), the final sample comprised 5,975 participants, of whom 648 reported a history of gallstones by themselves (as shown in Figure 1).

### Measurement of covariates

Demographics and lifestyle data came from the household interview questionnaires administered by highly trained medical personnel. Anthropometric indexes and biochemical parameters were obtained through medical examinations and subsequent laboratory assessments in the Mobile Examination Center (MEC). According to previous studies (5, 17), potential confounding factors correlated with gallstones and METS-VF were incorporated into the final analysis. The factors included demographic variables (age, height, race, blood pressure, gender, waist circumference (WC), educational attainment, weight, and physical activity). Total cholesterol (TC), uric acid (UA), FPG, albumin, low-density lipoprotein cholesterol (LDL-C), alanine aminotransferase (ALT), TG, gamma-glutamyl transferase (GGT), aspartate aminotransferase (AST), creatinine, and HDL-C were collected in blood samples. Questionnaire survey covered alcohol consumption, hypertension, diabetes mellitus, dietary intake factors, encompassing fat, energy, water, and sugar intake. All participants from 2013 to 2020 completed 24-h dietary recalls, and the mean consumption rates derived from these two recalls were utilizes. Detailed measurement methodologies and data acquisition for each variable can be accessed at www.cdc.gov/nchs/nhanes.

### Calculation formula of VAT surrogate markers

Various VAT and IR surrogate markers based on simple anthropometric measurements have been developed, such as metabolic score for insulin resistance (METS-IR), triglyceride-glucose index (TyG), METS-VF, lipid accumulation product (LAP) and visceral adiposity index (VAI).

METS-IR was calculated with the following formula (10, 17, 24–26):


$$\mathrm{METS}-IR=Ln\left[2\times FPG+TG\right]\times BMI/Ln\;\left[HDL-C\right]$$

$$TyG=Ln\;\left[TG\;\left(\mathrm{mg}/\mathrm{dL}\right)\times FPG\left(\mathrm{mg}/\mathrm{dL}\right)/2\right]$$

METS-VF was calculated with the following formula:


$$\begin{array}{l} \begin{array}{c} \mathrm{METS}-VF=3.239\times {\left[Ln\;\left(\mathrm{WHtR}\right)\right]}^{3}+0.011\times {\left[Ln\;\left(\mathrm{METS}-IR\right)\right]}^{3}\\ +0.319\times \mathrm{gender} \end{array}\\ \;\left(\mathrm{male}=1\mathrm{,female}=0\right)+4.466+0.594\times \left[Ln\;\left(\mathrm{Age}\right)\;\left(\mathrm{year}\right)\right] \end{array}$$

$$\mathrm{LAP}\;\left(\mathrm{male}\right)=TG*\left(WC-65\right)$$

$$\mathrm{LAP}\;\left(\mathrm{female}\right)=TG*\left(WC-58\right)$$

$$VAI\;\left(\mathrm{male}\right)=[WC/[\left(TG/1.03\right)\times \left(1.88\times BMI\right)])\times \left(1.31/HDL\right)+39.68]$$

$$VAI\;\left(\mathrm{female}\right)=[WC/[\left(TG/0.81\right)\times \left(1.89\times BMI\right)])\times \left(1.52/HDL\right)+36.58]$$

### Statistical analysis

METS-VF values were categorized into quartiles (Q1: ≤6.27; Q2: 6.27–6.69; Q3: 6.69–7.00; Q4: ≥7.00). Differences among quartile groups were assessed with chi-square test or Kruskal-Wallis H test. ORs and 95% CIs between gallstones and METS-VF were explored with multiple logistic regression models. The analysis incorporated three models: Model 1 (unadjusted), Model 2 (adjusted for race, gender, and age), and Model 3 (fully adjusted for drinking, educational level, TC, moderate physical activities, T2DM, albumin, SBP, DBP, ALT, AST, creatinine, GGT, total fat, total water, uric acid, total energy, and total sugar intake). The potential modifications of the correlation by covariates were explored through interaction tests and subgroup analyses. Furthermore, the non-linear correlation between gallstones and METS-VF was assessed through RCS analyses. Inflection point values were identified through the natural ratio test upon detecting non-linear correlation. Finally, the diagnostic efficacy of METS-VF, METS-IR, TyG, BMI, LAP, WHtR, and VAI in detecting was evaluated through ROC analyses. Data analyses were conducted with R software and Free Statistics software, with a significance threshold at *p* < 0.05 for all statistical tests.

## Results

### Clinical baseline features of subjects

Baseline demographic characteristics of the enrolled participants are detailed in Table 1, with attributes categorized according to gallstone status. Apart from drinking, liver functions, educational level, uric acid, TC, LDL-C, and dietary parameters (total sugar and water intake), significant differences in baseline characteristics were identified between the two cohorts. Individuals with gallstones demonstrated higher values in BMI, age, WC, FPG, TG, and METS-VF. Additionally, the proportion of females was significantly higher, and the prevalence of hypertension and T2DM was also higher in this group. Conversely, subjects with gallstones showed lower levels of albumin, creatinine, HDL-C, and total energy fat intake.

**Table 1:** Baseline characteristics of participants.

Characteristic	Non-stone formers	Stone formers	*p* value
Number	5,327	648	<0.001
Age, year	50.02 ± 17.42	57.67 ± 15.23	<0.001
Race, *n*%			<0.001
Mexican American	696 (13.1)	101 (15.6)	
Other Hispanic	501 (9.4)	83 (12.8)	
Non-Hispanic White	1761 (33.1)	258 (39.8)	
Non-Hispanic Black	1,359 (25.5)	105 (16.2)	
Other Race	1,010 (19)	101 (15.6)	
Moderate activities, *n*%			0.007
Yes	2,234 (41.9)	236 (36.4)	
No	3,093 (58.1)	412 (63.6)	
Diabetes, *n*%			<0.001
Yes	789 (15.3)	181 (28.8)	
No	4,363 (84.7)	448 (71.2)	
Hypertension			<0.001
Yes	1937 (36.4)	351 (54.2)	
No	3,381 (63.6)	297 (45.8)	
Education level, *n*%			0.357
Less than high school	1,021 (19.2)	134 (20.7)	
High school or above	4,306 (80.8)	514 (79.3)	
Drinking, *n*%			0.548
Current or ever, %	4,616 (86.7)	567 (87.5)	
Never	711 (13.3)	81 (12.5)	
Male, *n*%	2,706 (50.8)	184 (28.4)	<0.001
Weight, cm	82.33 ± 21.93	88.89 ± 23.44	<0.001
Body mass index, Kg/m2	29.37 ± 7.01	33.10 ± 8.16	<0.001
Height, cm	167.12 ± 10.02	163.71 ± 9.08	<0.001
Waist circumference, cm	99.78 ± 16.89	108.18 ± 16.86	<0.001
Systolic blood pressure, mmHg	125.50 ± 19.66	128.75 ± 20.76	0.019
Diastolic blood pressure, mmHg	72.30 ± 12.76	70.11 ± 14.88	0.017
FPG, mmol/L	6.26 ± 2.07	6.74 ± 2.32	<0.001
ALT, U/L	22.25 ± 18.50	21.89 ± 14.39	0.634
AST, U/L	21.92 ± 14.57	21.19 ± 10.44	0.215
GGT, U/L	32.25 ± 51.22	32.23 ± 40.96	0.994
Albumin, g/dl	4.02 ± 0.33	3.89 ± 0.35	<0.001
Creatinine, umol/L	75.00 (62.00, 88.00)	71.00 (59.00, 86.00)	<0.001
Uric acid, umol/L	321.20 (261.70, 380.70)	315.20 (267.70, 368.80)	0.325
Total cholesterol, mmol/L	4.80 ± 1.06	4.74 ± 1.17	0.199
Triglycerides, mmol/L	1.17 (0.84, 1.67)	1.40 (0.98, 1.84)	<0.001
HDL-cholesterol, mmol/L	1.39 ± 0.42	1.35 ± 0.36	0.006
LDL-cholesterol, mmol/L	2.84 ± 0.91	2.78 ± 1.03	0.153
PIR	2.61 ± 1.62	2.54 ± 1.51	0.310
Total sugar, g	87.48 (57.67, 127.44)	87.59 (57.98, 123.94)	0.797
Total energy, kcal	2037.58 ± 836.47	1892.53 ± 805.13	<0.001
Total fat, g	82.36 ± 39.00	77.81 ± 38.66	0.010
Total water, g	960.00 (450.00, 1618.28)	867.00 (445.88, 1567.50)	0.358
METS-VF	6.50 ± 0.64	6.88 ± 0.39	<0.001

Values are mean ± SD or number (%). P < 0.05 was deemed significant. BMI, body mass index; FPG, fasting blood glucose; TC, total cholesterol; TG, triglyceride; HDL-c, High density lipoprotein cholesterol; LDL-c, Low density lipoprotein cholesterol; GGT, glutamyl transpeptidase; METS-VF, metabolic score for visceral fat.

### The increase of METS-VF was positively correlated with the incidence of gallstones

**Figure 2:**
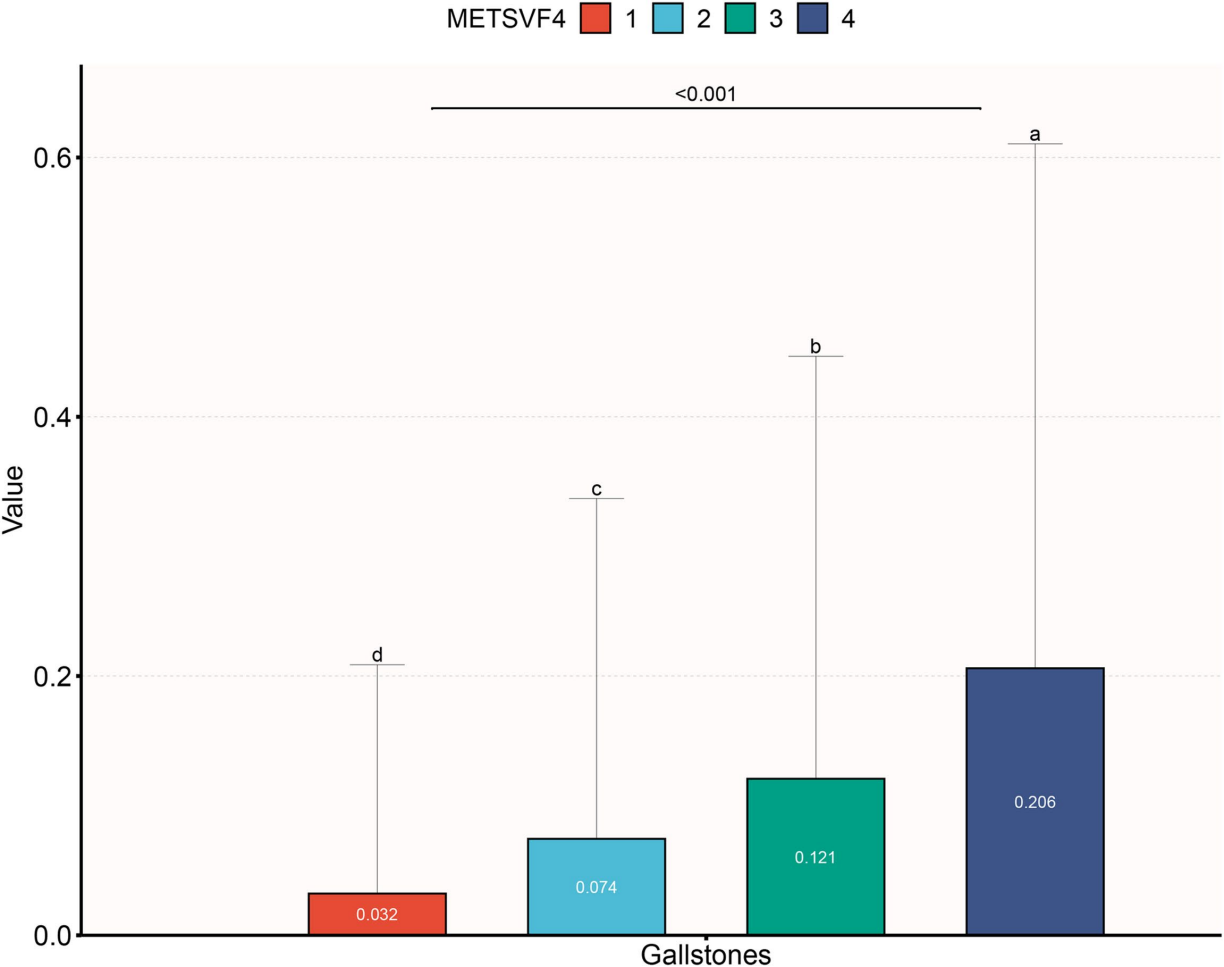
The prevalence of gallstones across quartiles of METS-VF.

As illustrated in Figure 2, the quartile range of METS-VF increased, with a notable rise in the prevalence of gallstones (3.2% vs. 7.4% vs. 12.1% vs. 20.6%, *p* < 0.001). In a fully adjusted model, each one-unit increase in METS-VF was correlated with a 2.075-fold higher risk of developing gallstones (OR = 3.075, 95% CI: 2.158, 4.381). According to the sensitivity analysis, METS-VF was categorized into quartiles, showing that in the fully adjusted Model 3, subjects in the second, third, and fourth quartiles exhibited a statistically significant increase in the risk of gallstones by 0.997, 1.702, and 2.363, respectively, compared to those in the lowest quartile (Table 2).

**Table 2:** Logistic regression analysis between METS-VF with gallbladder stone prevalence.

	Model1 OR (95% CI), *p* value	Model 2 OR (95% CI), *p* value	Model 3 OR (95% CI), *p* value
METS-VF	4.913 (3.945, 6.120), <0.001	4.066 (3.115, 5.307), <0.001	3.075 (2.158, 4.381), <0.001
METS-VF (Quartile)			
Q1	Reference	Reference	Reference
Q2	2.418 (1.71, 3.419), <0.001	2.246 (1.576, 3.199), <0.001	1.997 (1.291, 3.088), 0.002
Q3	4.13 (2.978, 5.728), <0.001	3.436 (2.428, 4.863), <0.001	2.702 (1.747, 4.178), <0.001
Q4	7.805 (5.704, 10.68), <0.001	5.901 (4.101, 8.492), <0.001	3.363 (2.080, 5.438), <0.001
*p* for trend	<0.001	<0.001	<0.001

Model 1: None covariates were adjusted; Model 2: gender, age and race were adjusted; Model 3: gender, age, race, drinking, educational level, TC, moderate physical activities, diabetes, albumin, SBP, DBP, ALT, AST, creatinine, GGT, uric acid, total water, total energy, total sugar and total fat were adjusted.

### Non-linearity analysis between METS-VF and gallstones

**Figure 3:**
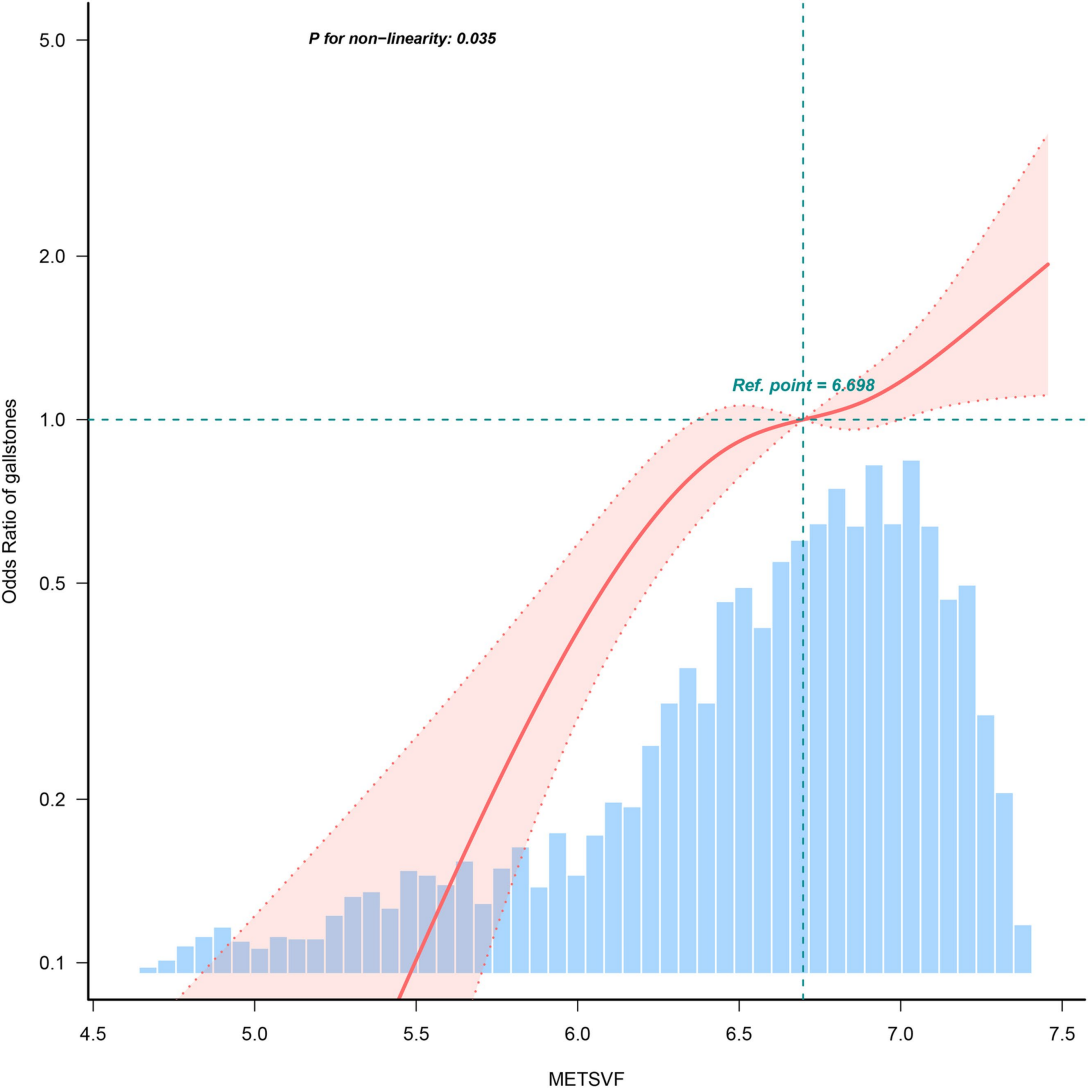
Restricted cubic spline fitting for the association between METS-VF levels and gallstones.

To further explore the correlation between METS-VF and gallstones, RCS analyses on Model 3 was conducted. The results depicted in Figure 3 revealed a non-linear correlation between METS-VF and gallstones. A subsequent threshold effect analysis detailed in Table 3 identified an inflection point for METS-VF at 6.698 (log-likelihood ratio < 0.001).

**Table 3:** Threshold effect analysis of METS-VF on gallbladder stone using the two-piecewise regression model.

METS-VF	Adjusted OR (95% CI)	*p* value
Inflection point	6.698	
METS-VF < 6.698	4.708 (2.368, 9.362)	<0.001
METS-VF > 6.698	2.780 (1.151, 6.714)	0.023
Log likelihood ratio		<0.001

METS-VF, metabolic score for visceral fat, statistically significant: *p* < 0.05. Gender, age, race, drinking, educational level, TC, moderate physical activities, diabetes, albumin, SBP, DBP, ALT, AST, creatinine, GGT, uric acid, total water, total energy, total sugar and total fat were adjusted.

### Subgroup analysis

**Figure 4:**
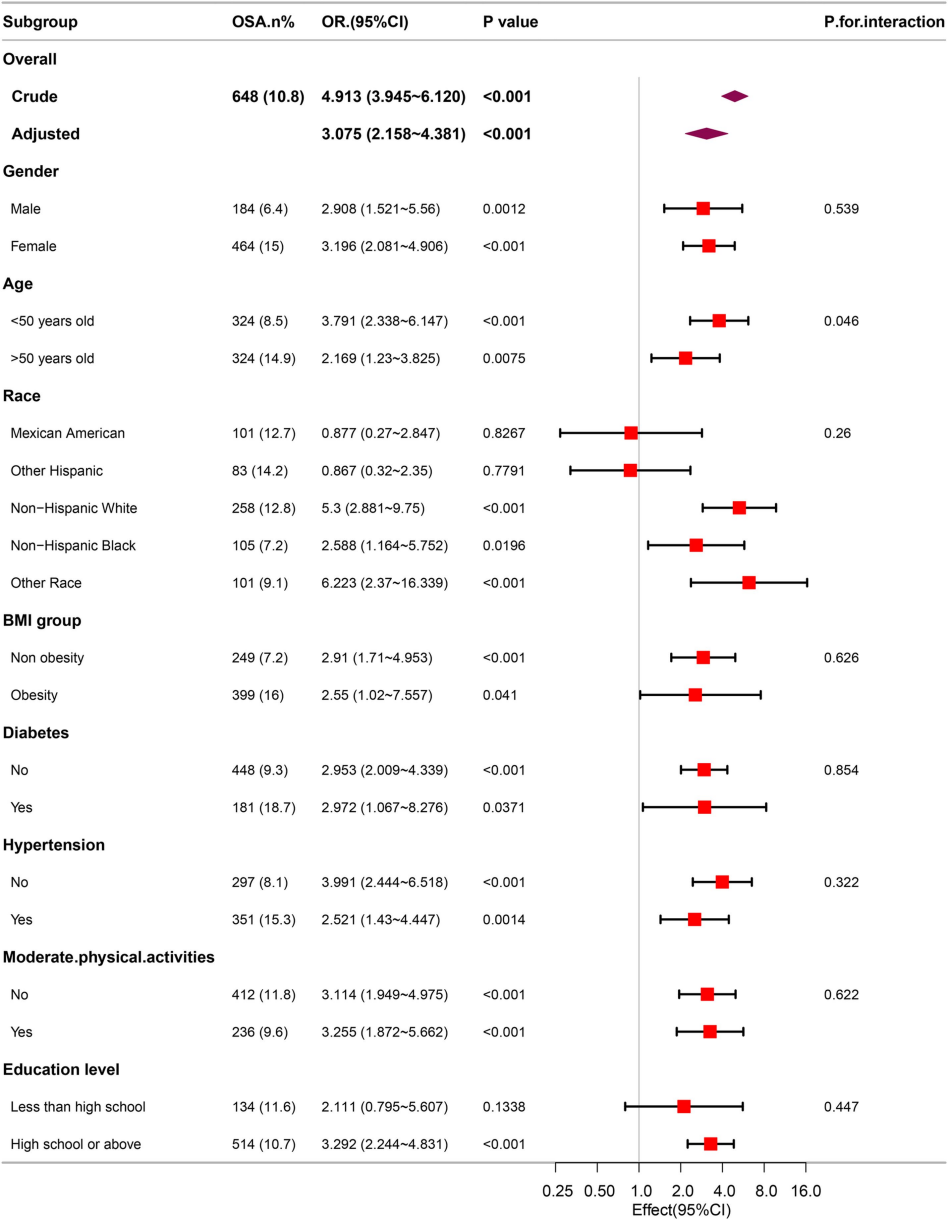
Association between METS-VF and the risk of gallstones in various subgroups.

To evaluate the robustness of the correlation between METS-VF and the prevalence of gallstones, subgroup analyses were performed. The results consistently demonstrated a notable correlation between METS-VF and gallstones within various subgroups (Figure 4). In the age subgroups, an elevated METS-VF was correlated with a higher prevalence of gallstones in the younger age subgroup.

### Predictive value of METS-VF for gallstones

**Figure 5:**
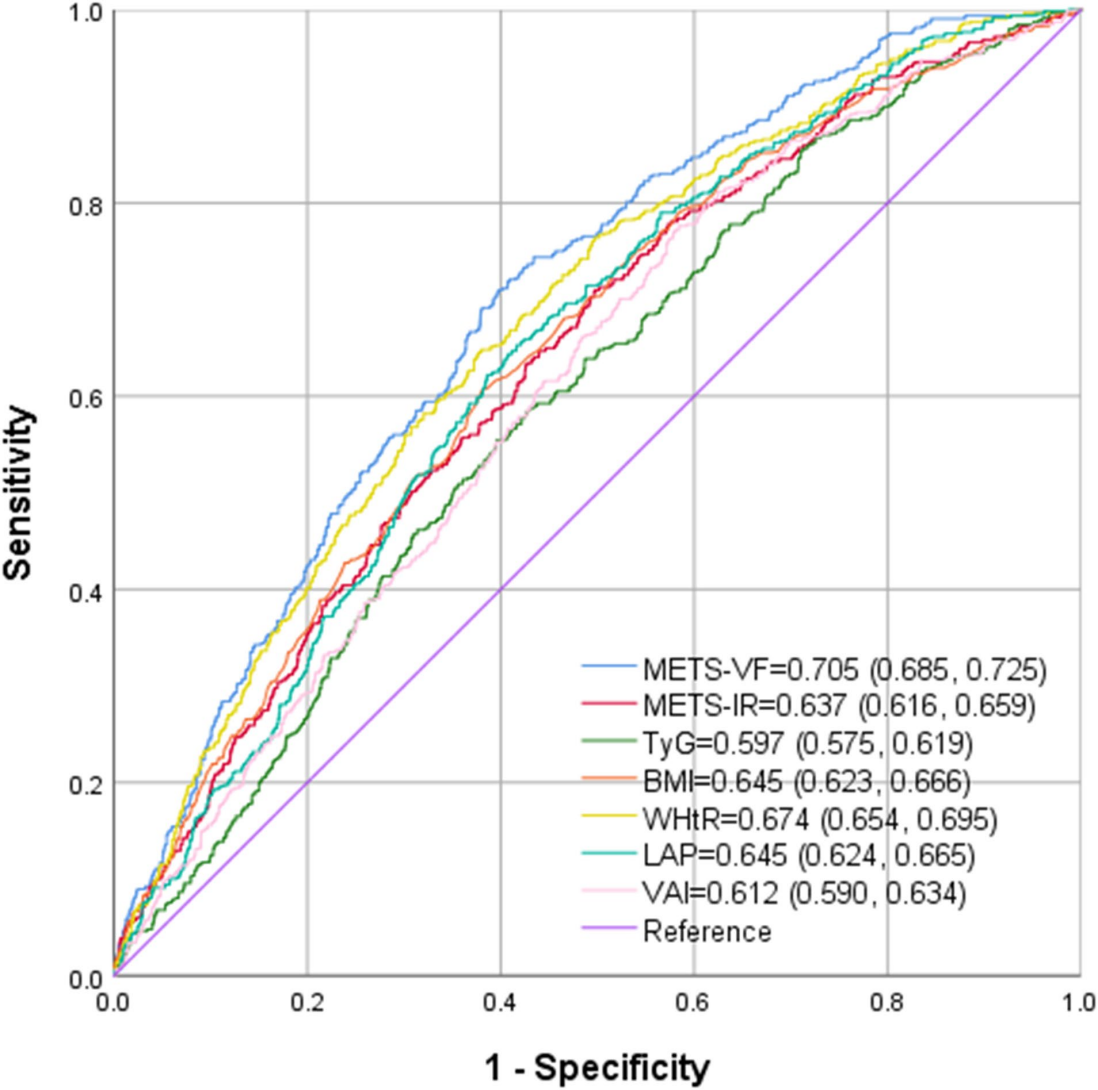
ROC analysis of METS-VF, METS-IR, BMI, WHtR, LAP and VAI to IR among American adults.

The ROC curve in Figure 5 presents the diagnostic performance of METS-VF, METS-IR, TyG, BMI, WHtR, LAP and VAI in identifying gallstones. As demonstrated in Table 4, METS-VF exhibited the highest diagnostic accuracy for gallstones, with an AUC value of 0.705 (95% CI: 0.685–0.725), significantly surpassing other VAT and IR surrogate markers (*p* < 0.001).

**Table 4:** The AUC for each index to discriminate gallbladder stone.

	AUC	95% CI	Cutoff value	Sensitivity	Specificity
METS-VF	0.705	0.685–0.725	6.767	0.731	0.610
METS-IR	0.637	0.616–0.659	42.06	0.710	0.502
TyG	0.597	0.575–0.619	8.71	0.585	0.574
BMI	0.645	0.623–0.666	30.15	0.607	0.619
WHtR	0.674	0.654–0.695	0.621	0.648	0.620
LAP	0.645	0.624–0.665	52.20	0.653	0.584
VAI	0.612	0.590–0.634	1.235	0.776	0.416

METS-VF, metabolic score for visceral fat; METS-IR, metabolic score for insulin resistance; TyG, triglyceride-glucose index; BMI, body mass index; WHtR, waist-to-height ratio; LAP, lipid accumulation product; VAI, visceral adiposity index.

## Discussion

This cross-sectional study encompassing 5,975 representative adults identified a notable positive correlation between METS-VF and gallstones. This correlation was particularly pronounced among younger individuals. Notably, non-linear correlation was observed between METS-VF and gallstones, with a saturation value of 6.698. Furthermore, among the seven indexes (WHtR, METS-VF, TyG, BMI, METS-IR, LAP, and VAI) evaluated, METS-VF demonstrated the largest AUC in predicting the odds of gallstones.

In recent years, there have been more and more scholars beginning to focus on the obesity and IR correlated with the development of gallstones. In a case–control study involving 881 subjects, HOMA-IR, a conventional index of IR, was found to correlate with developing gallstones (27), corroborating the findings from Wang et al. regarding the correlation between METS-IR and developing gallstones (10). Similarly, Wang et al. found that elevated triglyceride-glucose index, a novel indicator of IR, was correlated with the increased prevalence of gallstones (28). Furthermore, BMI, an index of overall adiposity, has been shown to double the risk of developing gallstones when individuals reach overweight or obese status (13, 29, 30). A Mendelian randomization study by Zhu et al. corroborated these findings by demonstrating an increased WC correlated with a heightened risk of developing gallstones (31). A reliable measure of central adiposity, WHtR, has also been identified in Taiwan and Iran as the most significant risk factor for developing gallstones among females (32, 33). However, the diagnostic utility of these indexes is constrained by the inability to distinctly differentiate between VAT and subcutaneous adipose tissue.

METS-VF is a novel VAT estimator recently developed by Bello-Chavolla et al. It has undergone a comprehensive validation and development, which has been documented in detail elsewhere (17). Due to the computational simplicity and high accuracy of METS-VF in predicting visceral obesity, increasing researchers have explored and corroborated the superior efficacy in assessing and forecasting the risk of diseases correlated with visceral obesity. In the studies, Yu et al. demonstrated that METS-VF exhibited a strong predictive capacity for CKD compared to alternative markers of central adiposity (18). Furthermore, METS-VF has shown applicability and reliability as a predictor of T2DM and hypertension in Chinese, outperforming other obesity evaluation indexes (34, 35). For non-obese females, METS-VF is instrumental in guiding the management and prevention of hyperuricemia (22). The correlation between METS-VF and gallstones, however, has not been studied to date. This study identified a significant and non-linear positive correlation between METS-VF and the prevalence of gallstones in a nationally representative sample for the first time. As a result of the ROC analysis, METS-VF possessed a significantly higher diagnostic value for gallstones than other VAT, IR surrogate markers, such as BMI, WHtR, VAI, LAP, METS-IR, TyG. These findings align with prior studies on METS-VF. These studies collectively supports the assertion that METS-VF is a superior predictive and diagnostic tool compared to traditional VAT surrogate indexes, with extensive potential applications in diseases correlated with visceral obesity.

Additionally, this study identified an age-related effect on the prevalence of gallstones through interaction testing. Consistent with the results, previous studies have demonstrated that the impact of obesity and metabolic syndrome on gallstones is more pronounced in younger individuals (36). The dietary patterns of younger individuals were typically characterized by the levels of calories, cholesterol, and fat, coupled with inadequate intake of dietary fiber. In addition, the rising incidence of obesity among this demographic was correlated with disruptions in lipid metabolism, thereby heightening the risk of gallstones formation (36–38). Furthermore, this is due to the fact that the prevalence of gallstones is already at a higher level in the older population and therefore its changes with METS-VF are flatter.

There are possible mechanistic explanations for the correlation between METS-VF and gallstones. (1) According to a study conducted in a high-risk Hispanic population, IR changes gallbladder function by increasing cholesterol-supersaturated bile production, which develop gallstones (27). Moreover, animal experiments have shown that mice with isolated hepatic IR are more likely to develop cholesterol gallstones (39). It is possible that the observed mechanism is correlated with increased expression of biliary cholesterol transporters, which is caused by the disinhibition of the forkhead transcription factor FoxO1. An alternative mechanism could involve hepatic IR, which diminishes the expression of bile acid synthetic enzymes, consequently producing a lithogenic bile salt profile. (2) There is a link between obesity and increased cholesterol secretion, leading to cholesterol-supersaturated bile precipitating as cholesterol gallstones (33). Gallstones may be developed in obese individuals due to impaired gallbladder motility for decreased sensitivity to cholecystokinin. (3) Through its regulation of bile acid metabolism, leptin, a hormone essential in the development of obesity, has been implicated in cholelithiasis development (40). (4) Rapid weight loss after metabolic bariatric surgery increasingly performed nowadays leads also to the development of gallstones in the long-term due to cholesterol supersaturation and reduced mobility of gallbladder (41).

The accuracy of transabdominal ultrasound in detecting gallstones is more than 95% (42). In this study, high METS-VF was found to be positively correlated with the risk of developing gallstones, particular for participants with METS-VF greater than 6.698. Therefore, transabdominal ultrasound testing is necessary to screen for gallstones in participants with METS-VF greater than 6.698.

### Study strengths and limitations

The study’s primary strength is its distinction as the first cross-sectional analysis to explore the correlation between METS-VF and gallstones, supported by a sufficiently large and representative sample size. However, this study really had limitations that should be acknowledged. Firstly, cross-sectional studies are limited in their ability to establish causality, leaving the causal correlation between METS-VF and gallstones, as well as the directionality of this potential correlation, to be elucidated through further research. Secondly, a notable limitation of this study is the reliance on self-reported diagnoses of gallstones by subjects, which introduces an inherent recall bias. Consequently, future prospective studies are warranted to address these limitations. Thirdly, there are numerous potential influencing factors for METS-VF and gallstones. Despite the model’s inclusion of as many pertinent covariates as possible, it remains challenging to completely eliminate the influence of other variables, such as blood disorders, previous bariatric surgeries and genetic factors.

## Conclusion

In conclusion, the present study identified a significant correlation between elevated METS-VF and an increased prevalence of gallstones. Compared to other indexes, METS-VF emerges as a more convenient and effective surrogate marker for VAT measurement. It holds potential for personalizing interventions and aiding physicians in identifying populations that may benefit from gallstone screening, thereby reducing both the economic burden and the risk of serious complications correlated with gallstones.

## Data Availability

Publicly available datasets were analyzed in this study. This data can be found at: NHANES, http://www.cdc.gov/nhanes.

## Footnotes

^1^www.cdc.gov/nchs/nhanes

## References

[1] Friedman GD. Natural history of asymptomatic and symptomatic gallstones. Am J Surg. (1993) 165:399–404. doi: 10.1016/S0002-9610(05)80930-4 8480871

[2] Hundal R Shaffer EA. Gallbladder cancer: epidemiology and outcome. Clin Epidemiol. (2014) 6:99–109. doi: 10.2147/CLEP.S37357, PMID: 24634588 PMC3952897

[3] Stinton LM Shaffer EA. Epidemiology of gallbladder disease: cholelithiasis and cancer. Gut Liver. (2012) 6:172–87. doi: 10.5009/gnl.2012.6.2.172, PMID: 22570746 PMC3343155

[4] Figueiredo JC Haiman C Porcel J Buxbaum J Stram D Tambe N. Sex and ethnic/racial-specific risk factors for gallbladder disease. BMC Gastroenterol. (2017) 17:153. doi: 10.1186/s12876-017-0678-6, PMID: 29221432 PMC5723039

[5] Shaffer EA. Gallstone disease: epidemiology of gallbladder stone disease. Best Pract Res Clin Gastroenterol. (2006) 20:981–96. doi: 10.1016/j.bpg.2006.05.004 17127183

[6] Marschall HU Krawczyk M Grunhage F Katsika D Einarsson C Lammert F. Gallstone disease in Swedish twins is associated with the Gilbert variant of UGT1A1. Liver Int. (2013) 33:904–8. doi: 10.1111/liv.12141, PMID: 23517300

[7] Tanaka H Imasato M Yamazaki Y Matsumoto K Kunimoto K Delpierre J. Claudin-3 regulates bile canalicular paracellular barrier and cholesterol gallstone core formation in mice. J Hepatol. (2018) 69:1308–16. doi: 10.1016/j.jhep.2018.08.025 30213590

[8] Zhu Q Sun X Ji X Zhu L Xu J Wang C. The association between gallstones and metabolic syndrome in urban Han Chinese: a longitudinal cohort study. Sci Rep. (2016) 6:29937. doi: 10.1038/srep29937, PMID: 27443986 PMC4957232

[9] Mulita F Tchabashvili L Bousis D Kehagias D Kaplanis C Liolis E. Gallstone ileus: a rare cause of small intestine obstruction. Clin Case Rep. (2021) 9:e04924. doi: 10.1002/ccr3.4924, PMID: 34765198 PMC8572328

[10] Wang J Yang J Chen Y Rui J Xu M Chen M. Association of METS-IR index with prevalence of gallbladder stones and the age at the first gallbladder stone surgery in US adults: a cross-sectional study. Front Endocrinol (Lausanne). (2022) 13:1025854. doi: 10.3389/fendo.2022.1025854, PMID: 36263324 PMC9574223

[11] Paschos P Paletas K. Non alcoholic fatty liver disease and metabolic syndrome. Hippokratia. (2009) 13:9–19. PMID: 19240815 PMC2633261

[12] Aune D Norat T Vatten LJ. Body mass index, abdominal fatness and the risk of gallbladder disease. Eur J Epidemiol. (2015) 30:1009–19. doi: 10.1007/s10654-015-0081-y, PMID: 26374741

[13] Banim PJ Luben RN Bulluck H Sharp SJ Wareham NJ Khaw KT. The aetiology of symptomatic gallstones quantification of the effects of obesity, alcohol and serum lipids on risk. Epidemiological and biomarker data from a UK prospective cohort study (EPIC-Norfolk). Eur J Gastroenterol Hepatol. (2011) 23:733–40. doi: 10.1097/MEG.0b013e3283477cc9, PMID: 21623190

[14] Koenen M Hill MA Cohen P Sowers JR. Obesity, adipose tissue and vascular dysfunction. Circ Res. (2021) 128:951–68. doi: 10.1161/CIRCRESAHA.121.318093, PMID: 33793327 PMC8026272

[15] Stefan N. Causes, consequences, and treatment of metabolically unhealthy fat distribution. Lancet Diabetes Endocrinol. (2020) 8:616–27. doi: 10.1016/S2213-8587(20)30110-8, PMID: 32559477

[16] Sorimachi H Obokata M Takahashi N Reddy YNV Jain CC Verbrugge FH. Pathophysiologic importance of visceral adipose tissue in women with heart failure and preserved ejection fraction. Eur Heart J. (2021) 42:1595–605. doi: 10.1093/eurheartj/ehaa823, PMID: 33227126 PMC8060057

[17] Bello-Chavolla OY Antonio-Villa NE Vargas-Vazquez A Viveros-Ruiz TL Almeda-Valdes P Gomez-Velasco D. Metabolic score for visceral fat (METS-VF), a novel estimator of intra-abdominal fat content and cardio-metabolic health. Clin Nutr. (2020) 39:1613–21. doi: 10.1016/j.clnu.2019.07.012, PMID: 31400997

[18] Yu P Meng X Kan R Wang Z Yu X. Association between metabolic scores for visceral fat and chronic kidney disease: a cross-sectional study. Front Endocrinol (Lausanne). (2022) 13:1052736. doi: 10.3389/fendo.2022.1052736, PMID: 36545336 PMC9762045

[19] Antonio-Villa NE Bello-Chavolla OY Vargas-Vazquez A Mehta R Aguilar-Salinas CA. Metabolic syndrome study G: the combination of insulin resistance and visceral adipose tissue estimation improves the performance of metabolic syndrome as a predictor of type 2 diabetes. Diabet Med. (2020) 37:1192–201. doi: 10.1111/dme.14274, PMID: 32061103

[20] Feng Y Yang X Li Y Wu Y Han M Qie R. Metabolic score for visceral fat: a reliable indicator of visceral obesity for predicting risk for hypertension. Nutrition. (2022) 93:111443. doi: 10.1016/j.nut.2021.111443, PMID: 34563934

[21] Feng L Chen T Wang X Xiong C Chen J Wu S. Metabolism score for visceral fat (METS-VF): a new predictive surrogate for CKD risk. Diabetes Metab Syndr Obes. (2022) 15:2249–58. doi: 10.2147/DMSO.S370222 35936056 PMC9346409

[22] Liu XZ Chen DS Xu X Li HH Liu LY Zhou L. Longitudinal associations between metabolic score for visceral fat and hyperuricemia in non-obese adults. Nutr Metab Cardiovasc Dis. (2020) 30:1751–7. doi: 10.1016/j.numecd.2020.06.001, PMID: 32811739

[23] Zipf G Chiappa M Porter KS Ostchega Y Lewis BG Dostal J. National health and nutrition examination survey: plan and operations, 1999-2010. Vital Health Stat. (2013) 1:1–37. PMID: 25078429

[24] Zhang Q Xiao S Jiao X Shen Y. The triglyceride-glucose index is a predictor for cardiovascular and all-cause mortality in CVD patients with diabetes or pre-diabetes: evidence from NHANES 2001-2018. Cardiovasc Diabetol. (2023) 22:279. doi: 10.1186/s12933-023-02030-z, PMID: 37848879 PMC10583314

[25] Kahn HS. The "lipid accumulation product" performs better than the body mass index for recognizing cardiovascular risk: a population-based comparison. BMC Cardiovasc Disord. (2005) 5:26. doi: 10.1186/1471-2261-5-26, PMID: 16150143 PMC1236917

[26] Deng H Hu P Li H Zhou H Wu X Yuan M. Novel lipid indicators and the risk of type 2 diabetes mellitus among Chinese hypertensive patients: findings from the Guangzhou heart study. Cardiovasc Diabetol. (2022) 21:212. doi: 10.1186/s12933-022-01660-z, PMID: 36243748 PMC9571423

[27] Nervi F Miquel JF Alvarez M Ferreccio C Garcia-Zattera MJ Gonzalez R. Gallbladder disease is associated with insulin resistance in a high risk Hispanic population. J Hepatol. (2006) 45:299–305. doi: 10.1016/j.jhep.2006.01.026, PMID: 16516330

[28] Wang J Li H Hu J Shi R Qin C Chen X. Relationship of triglyceride-glucose index to gallstone prevalence and age at first gallstone surgery in American adults. Sci Rep. (2024) 14:16749. doi: 10.1038/s41598-024-67883-0, PMID: 39033195 PMC11271289

[29] Katsika D Tuvblad C Einarsson C Lichtenstein P Marschall HU. Body mass index, alcohol, tobacco and symptomatic gallstone disease: a Swedish twin study. J Intern Med. (2007) 262:581–7. doi: 10.1111/j.1365-2796.2007.01860.x, PMID: 17908165

[30] Zhang M Bai Y Wang Y Cui H Zhang W Zhang L. Independent association of general and central adiposity with risk of gallstone disease: observational and genetic analyses. Front Endocrinol (Lausanne). (2024) 15:1367229. doi: 10.3389/fendo.2024.1367229, PMID: 38529389 PMC10961427

[31] Zhu Q Xing Y Fu Y Chen X Guan L Liao F. Causal association between metabolic syndrome and cholelithiasis: a Mendelian randomization study. Front Endocrinol (Lausanne). (2023) 14:1180903. doi: 10.3389/fendo.2023.1180903, PMID: 37361524 PMC10288183

[32] Radmard AR Merat S Kooraki S Ashraf M Keshtkar A Sharafkhah M. Gallstone disease and obesity: a population-based study on abdominal fat distribution and gender differences. Ann Hepatol. (2015) 14:702–9. doi: 10.1016/S1665-2681(19)30765-3, PMID: 26256899

[33] Chou TS Lin CL Chen LW Hu CC Chang JJ Yen CL. Waist-to-height ratio for the prediction of gallstone disease among different obesity indicators. Obes Sci Pract. (2023) 9:30–41. doi: 10.1002/osp4.650, PMID: 36789027 PMC9913192

[34] Feng Y Yang X Li Y Wu Y Han M Qie R. Metabolic score for visceral fat: a novel predictor for the risk of type 2 diabetes mellitus. Br J Nutr. (2022) 128:1029–36. doi: 10.1017/S0007114521004116, PMID: 34632975

[35] Zhang F Wang Y Zhou J Yu L Wang Z Liu T. Association between metabolic score for visceral fat and the risk of hypertension in different ethnic groups: a prospective cohort study in Southwest China. Front Endocrinol (Lausanne). (2024) 15:1302387. doi: 10.3389/fendo.2024.1302387, PMID: 38562413 PMC10982387

[36] Su PY Hsu YC Cheng YF Kor CT Su WW. Strong association between metabolically-abnormal obesity and gallstone disease in adults under 50 years. BMC Gastroenterol. (2019) 19:117. doi: 10.1186/s12876-019-1032-y, PMID: 31272395 PMC6610843

[37] Yuan S Gill D Giovannucci EL Larsson SC. Obesity, type 2 diabetes, lifestyle factors, and risk of gallstone disease: a Mendelian randomization investigation. Clin Gastroenterol Hepatol. (2022) 20:e529–37. doi: 10.1016/j.cgh.2020.12.034, PMID: 33418132

[38] Cheng J Zhuang Q Wang W Li J Zhou L Xu Y. Association of pro-inflammatory diet with increased risk of gallstone disease: a cross-sectional study of NHANES January 2017-march 2020. Front Nutr. (2024) 11:1344699. doi: 10.3389/fnut.2024.1344699, PMID: 38549748 PMC10972905

[39] Biddinger SB Haas JT Yu BB Bezy O Jing E Zhang W. Hepatic insulin resistance directly promotes formation of cholesterol gallstones. Nat Med. (2008) 14:778–82. doi: 10.1038/nm1785, PMID: 18587407 PMC2753607

[40] Wen J Jiang Y Lei Z He J Ye M Fu W. Leptin influence Cholelithiasis formation by regulating bile acid metabolism. Turk J Gastroenterol. (2021) 32:97–105. doi: 10.5152/tjg.2020.19594, PMID: 33893772 PMC8975486

[41] Kehagias I Bellou A Kehagias D Markopoulos G Amanatidis T Alexandrou A. Long-term (11 + years) efficacy of sleeve gastrectomy as a stand-alone bariatric procedure: a single-center retrospective observational study. Langenbeck's Arch Surg. (2022) 408:4. doi: 10.1007/s00423-022-02734-y 36577828

[42] Sebghatollahi V Parsa M Minakari M Azadbakht S. A clinician's guide to gallstones and common bile duct (CBD): a study protocol for a systematic review and evidence-based recommendations. Health Sci Rep. (2023) 6:e1555. doi: 10.1002/hsr2.1555, PMID: 37706014 PMC10496460

